# Moxifloxacin and bilateral acute iris transillumination

**DOI:** 10.1186/1869-5760-3-10

**Published:** 2013-01-14

**Authors:** Robert M Knape, Fouad E Sayyad, Janet L Davis

**Affiliations:** 1 Bascom Palmer Eye Institute, University of Miami Miller School of Medicine, 900 N.W. 17th St, Miami, FL 33136, USA

**Keywords:** Moxifloxacin, Fluoroquinolone, Uveitis, Iris transillumination, Pigment dispersion

## Abstract

Recent publications have alerted clinicians to a syndrome of uveitic transilluminating iris depigmentation associated with systemic fluoroquinolones and other antibiotics. Bilateral acute iris transillumination, which is associated with loss of the iris pigment epithelium and results in iris transillumination, differs from the previously described bilateral acute depigmentation of the iris, which is associated with atrophy of the iris stroma without transillumination. We present a case of fluoroquinolone-associated uveitis with anterior segment optical coherence tomography imaging to highlight some observations about this syndrome. We interpret pharmacokinetic data to help explain why oral, but not topical, moxifloxacin may cause fluoroquinolone-associated uveitis.

## 

Dear editor:

Recent publications have alerted clinicians to a syndrome of transilluminating iris depigmentation associated with the use of systemic fluoroquinolones and other antibiotics [1,2]. This syndrome, termed bilateral acute iris transillumination by some authors [1], differs from the previously described bilateral acute depigmentation of the iris, which causes reversible atrophy of the iris stroma without iris transillumination [3]. In contrast, fluoroquinolone-associated uveitis preferentially targets the iris pigment epithelium, leading to irreversible iris transillumination [2].

We present a case of fluoroquinolone-associated uveitis with anterior segment optical coherence tomography (OCT) imaging to highlight some observations about this syndrome. We interpret pharmacokinetic data to help explain why oral, but not topical, moxifloxacin may cause fluoroquinolone-associated uveitis. We also discuss the possible impact of the presence of the native crystalline lens. Finally, we note that the anterior segment OCT findings suggest that both the iris stroma and iris pigment epithelium are affected in fluoroquinolone-associated uveitis.

## Report of a case

**Figure 1:**
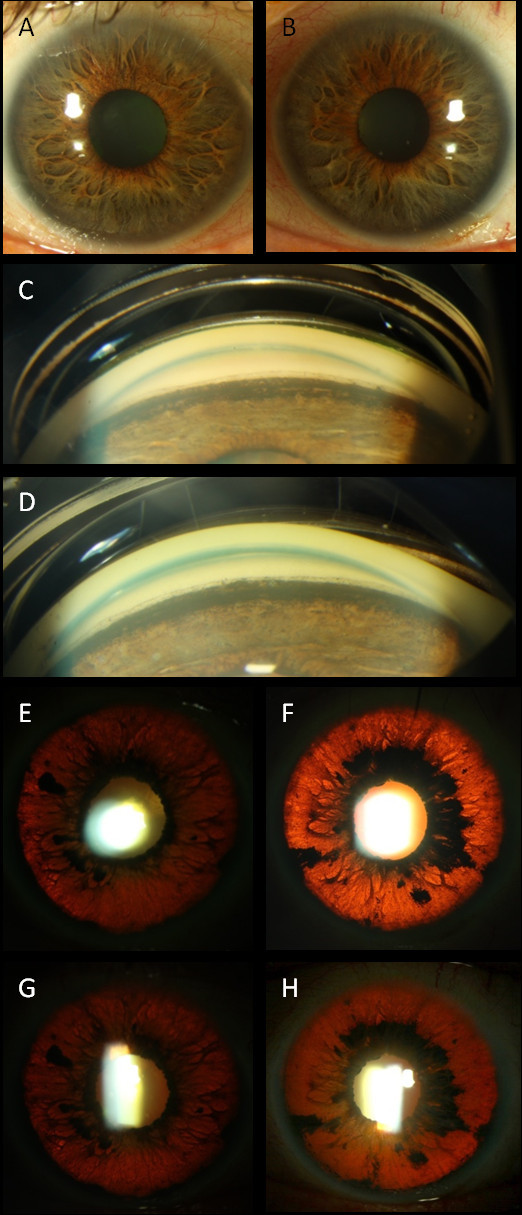
**Clinical presentation of the anterior segment.** Pigmented cells in the anterior chamber with deposition of a pigmented ‘wreath’ on the corneal endothelium adjacent to the limbus are seen in the right (**A**) and left (**B**) eyes. Gonioscopy shows pigment anterior to Schwalbe's line and extending on to the peripheral iris in the right (**C**) and left (**D**) eyes. Transillumination defects noted at presentation in the right (**E**) and left (**F**) eyes were stable 2 weeks later in the right (**G**) and left (**H**) eyes.

**Figure 2:**
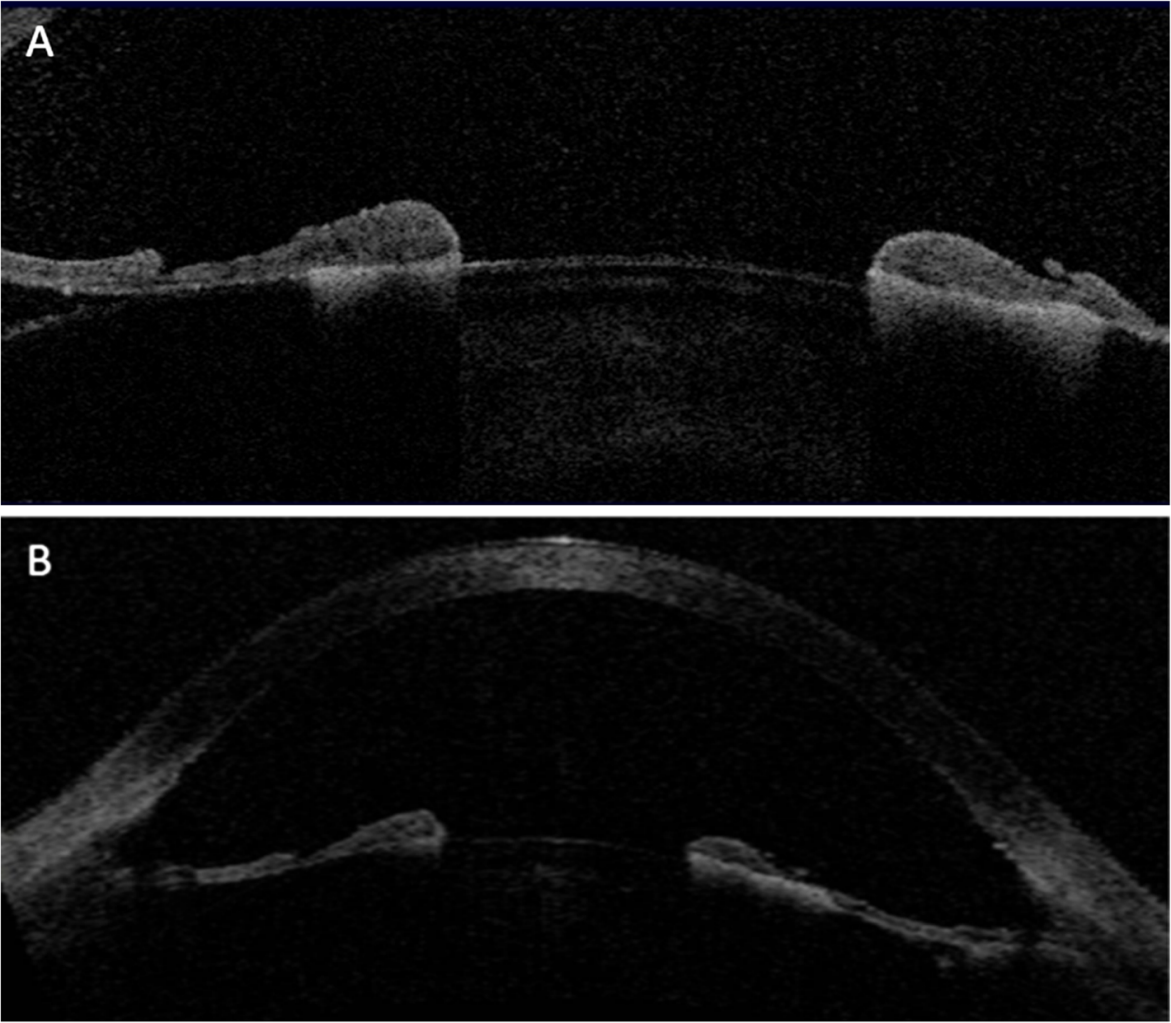
**OCT of the anterior segment.** The right (**A**) and left (**B**) eyes show significant stromal thinning with iris concavity and posterior synechiae.

A 70-year-old Caucasian man complained of blurry vision and photophobia in both eyes for 2 weeks. His past medical history was notable for lung cancer and an episode of pneumonia treated with oral moxifloxacin 3 days before the ocular symptoms began. His visual acuity was 20/30 OD and 20/40 OS with intraocular pressures (IOP) of 35 mmHg in both eyes. He had mild corneal edema and symmetric 3+ anterior chamber pigmented cells (Figure 1A,B). Gonioscopy revealed dense pigment obscuring all angle structures (Figure 1C,D). The posterior segments were normal. Serum laboratory evaluation and polymerase chain reaction of the right eye aqueous humor were negative for HSV, VZV, and CMV. He was treated with topical steroids and cycloplegic and IOP-lowering medications with little change in vision, IOP, or pupil size. Retroillumination images at presentation and 2 weeks later showed stable iris transillumination defects (Figure 1E,F,G,H), suggesting an acute, self-limited injury. Anterior segment OCT images of the iris demonstrated thinning, concavity, and adherence to the lens (Figure 2A,B). The patient died from respiratory failure after his 2-week follow-up visit.

## Comment

Previous reports have noted fluoroquinolone-associated uveitis only with systemic fluoroquinolones [1,2]. Pharmacokinetic data may explain why topical administration has not been associated with this clinical presentation. After topical administration, there is a greater-than-tenfold higher concentration of moxifloxacin in aqueous (2.28 ± 1.23 μg/mL) than in vitreous (0.11 ± 0.05 μg/mL) [4], whereas oral administration produces similar aqueous (1.34 ± 0.66 μg/mL) and vitreous concentrations (1.58 ± 0.80 μg/mL) [5]. The steady serum and vitreous reservoirs of moxifloxacin during oral administration may maintain drug levels in the tissue at risk better than intermittent topical therapy.

The lens status of affected patients may also be relevant. To our knowledge, only phakic patients have been reported with fluoroquinolone-associated uveitis [1,2]. Less drug diffuses posteriorly in phakic eyes [6], and posterior-to-anterior clearance may also be impaired in phakic eyes. Trapping of drug in the posterior chamber by synechiae between an intact lens and the iris, as demonstrated in our OCT (Figure 2), may result in higher drug concentrations adjacent to the posterior layers of the iris.

Why is intravitreal moxifloxacin injection apparently safe [7]? Experience gained from cidofovir-induced uveitis and hypotony indicates that toxicity to the nonpigmented epithelium of the ciliary body is uncommon after intravitreal therapy [8] compared to systemic administration [9], an effect that may be due to the more rapid clearance of intravitreally administered drug compared to recirculation from a serum depot. Additionally, *in vitro* toxicity studies have not examined the effect on the iris pigment epithelium [10], which may have different susceptibility to moxifloxacin toxicity. Finally, greater light exposure of the iris may have a bearing on the achieved toxicity.

Clinicians confronted with patients with acute onset of uveitis and pigment dispersion should be aware of the association between systemic fluoroquinolones and uveitis. Treatment with corticosteroids for prolonged pigment dispersion after the initial inflammatory phase is likely unnecessary and may contribute to glaucoma in steroid responders. Intraocular pressure would be predicted to improve as the load of pigment in the trabecular meshwork subsides.

## Competing interests

The authors declare that they have no competing interests.

## Authors’ contributions

RK, FS, and JD confirm that all authors made a direct and substantial contribution to the report by conceiving and designing the study, analyzing and interpreting the findings, writing the manuscript or providing critical revisions, and reading and approving the final version of the manuscript.

## References

[B1] Tugal-Tutkun I Onal S Garip A Taskapili M Kazokoglu H Kadayifcilar S Kestelyn P Bilateral acute iris transillumination Arch Ophthalmol 2011 129 10 1312 1319 10.1001/archophthalmol.2011.310 21987674

[B2] Hinkle DM Dacey MS Mandelcorn E Kalyani P Mauro J Bates JH Soukasian SH Holland GN Foster CS Fraunfelder FT Davis JL Fraunfelder FW Bilateral uveitis associated with fluoroquinolone therapy Cutan Ocul Toxicol 2012 31 2 111 116 10.3109/15569527.2011.617024 21981449

[B3] Tugal-Tutkun I Araz B Taskapili M Akova YA Yalniz-Akkaya Z Berker N Emre S Gezer A Bilateral acute depigmentation of the iris: report of 26 new cases and four-year follow-up of two patients Ophthalmology 2009 116 8 1552 1557 10.1016/j.ophtha.2009.02.019 19545903

[B4] Hariprasad SM Blinder KJ Shah GK Apte RS Rosenblatt B Holekamp NM Thomas MA Mieler WF Chi J Prince RA Penetration pharmacokinetics of topically administered 0.5 % moxifloxacin ophthalmic solution in human aqueous and vitreous Arch Ophthalmol 2005 123 1 39 44 10.1001/archopht.123.1.39 15642810

[B5] Hariprasad SM Shah GK Mieler WF Feiner L Blinder KJ Holekamp NM Gao H Prince RA Vitreous and aqueous penetration of orally administered moxifloxacin in humans Arch Ophthalmol 2006 124 2 178 182 10.1001/archopht.124.2.178 16476886

[B6] Fuller JJ McGwin G Jr Phakic status affects vitreous penetration of topical moxifloxacin Arch Ophthalmol 2006 124 5 749 author reply 749 16682605 10.1001/archopht.124.5.749-a

[B7] Bennett MD Yee W Bryan JS Pegaptanib combined with intravitreal injection of moxifloxacin as treatment of wet macular degeneration Retina 2008 28 7 976 980 10.1097/IAE.0b013e3181733733 18698300

[B8] Taskintuna I Rahhal FM Rao NA Wiley CA Mueller AJ Banker AS De Clercq E Arevalo JF Freeman WR Adverse events and autopsy findings after intravitreous cidofovir (HPMPC) therapy in patients with acquired immune deficiency syndrome (AIDS) Ophthalmology 1997 104 11 1827 1836 discussion 1836–1837 9373113 10.1016/s0161-6420(97)30020-7

[B9] Akler ME Johnson DW Burman WJ Johnson SC Anterior uveitis and hypotony after intravenous cidofovir for the treatment of cytomegalovirus retinitis Ophthalmology 1998 105 4 651 657 10.1016/S0161-6420(98)94019-2 9544639

[B10] Kernt M Neubauer AS Ulbig MW Kampik A Welge-Lüssen U In vitro safety of intravitreal moxifloxacin for endophthalmitis treatment J Cataract Refract Surg 2008 34 3 480 488 10.1016/j.jcrs.2007.10.046 18299076

